# Research trends and hotspots in the tumor microenvironment of ovarian cancer: a bibliometrics and visualization study from 2005 to 2024

**DOI:** 10.3389/fimmu.2025.1605695

**Published:** 2025-08-28

**Authors:** Jingwen Wang, Guohao Yin, Fangyu Hou, Xiangyu Yin, Tao Liu

**Affiliations:** ^1^ Center for Reproductive Medicine, Department of Obstetrics and Gynecology, QiLu Hospital of Shandong University, Jinan, Shandong, China; ^2^ Center for Reproductive Medicine, Department of Obstetrics and Gynecology, Affiliated Hospital of Jining Medical University, Jining, Shandong, China; ^3^ Department of Clinical Medicine, Jining Medical University, Jining, Shandong, China; ^4^ Hepatobiliary Surgery, Affiliated Hospital of Jining Medical University, Jining, Shandong, China

**Keywords:** ovarian cancer, tumor microenvironment, bibliometrics, visualization, research trends, research hotspots

## Abstract

**Background:**

The tumor microenvironment (TME) is crucial in influencing the progression and therapeutic response of ovarian cancer.

**Method:**

This study conducted a comprehensive bibliometric and visualization analysis of research trends and focal areas concerning the ovarian cancer microenvironment from 2005 to 2024. A total of 1,720 pertinent articles were identified from the Web of Science Core Collection (WoSCC) database.

**Results:**

The analysis revealed a notable increase in research activity on the TME of ovarian cancer, particularly during the period from 2019 to 2022. The United States and China were the most active countries in this field, and the University of Texas System was the scientific research institution with the largest number of articles. *Cancer Research* and *Clinical Cancer Research* were the most cited journals. Weiping Zou and Anil K Sood were recognized as the most cited researchers. The study systematically identified key research hotspots within this field, encompassing immune checkpoint inhibitors, single-cell RNA sequencing technology, the TME heterogeneity, the TGFβ signaling pathway, and the impact of PARP inhibitors on the tumor immune microenvironment (TIME).

**Conclusion:**

This study provides a valuable reference for the evolution and prospective directions of TME research in ovarian cancer, underscoring the critical importance of a comprehensive understanding of the TME to enhance treatment strategies for ovarian cancer.

## 1 Introduction

Ovarian cancer is an aggressive and fatal malignant tumor that poses a significant threat to women’s health. It is characterized by late detection, high recurrence rate, and limited treatment options. The pathogenesis of ovarian cancer is intricate and multifactorial, with the tumor microenvironment (TME) playing a crucial role in its progression (1). Studies have shown that the TME comprises not only tumor cells but also a diverse array of non-tumor components, including cancer-associated fibroblasts (CAFs), immune cells, endothelial cells, and the extracellular matrix. These components interact in complex ways to influence tumor initiation, progression, and metastasis (2–4). For instance, CAFs are recognized as pivotal regulators within the TME of ovarian cancer, facilitating tumor cell proliferation, migration, and angiogenesis through the secretion of growth factors and cytokines (4, 5). Moreover, immune cells within the TME, such as myeloid-derived suppressor cells (MDSCs), significantly contribute to tumor progression by suppressing the anti-tumor immune response (6, 7). Consequently, targeting the TME has emerged as a crucial focus in ovarian cancer research (8).

The mechanisms underlying the TME in ovarian cancer remain incompletely understood. Despite significant advancements in recent years, numerous aspects require further investigation, including the crosstalk between various cell types, the roles of specific signaling pathways, and the impact of the TME on therapeutic response. Studies have demonstrated that the TME plays a crucial role in the progression of ovarian cancer and the development of chemotherapy resistance, particularly concerning CAFs and TAMs (9–11). Furthermore, the complexity of the TME makes its role in tumor biology more important, especially in the interaction between tumor cells and the surrounding matrix (12). For instance, research indicates that the chronic inflammatory response within the TME is intricately linked to tumor initiation and progression, potentially influencing tumor growth and metastasis (13). Understanding the relationship between the TME and the pathogenesis of ovarian cancer is crucial to developing effective therapeutic strategies. Targeting specific components of the TME may disrupt the growth and survival mechanisms of tumors, augment the immune response against the tumor, and ultimately enhance patient prognosis. Nonetheless, additional research is required to elucidate the intricate interactions within the TME and their impact on the initiation and progression of ovarian cancer.

Numerous narrative reviews and meta-analyses have provided valuable insights into the research domain of the ovarian cancer TME, including the roles of specific cell types such as CAFs and tumor-associated macrophages (TAMs) (14, 15), the elucidation of particular signaling pathways (16), or advancements in immunotherapy (17). Nevertheless, these approaches are limited in capturing the broader landscape of the field. For instance, narrative reviews may be influenced by the author’s subjective selection and interpretation, potentially introducing personal bias. Additionally, these reviews may struggle to objectively quantify and visualize the evolution of research priorities, collaboration networks, and emerging trends over extended periods. Meta-analyses are typically employed to synthesize quantitative outcomes from comparable clinical trials or specific experimental interventions, focusing on particular aspects of a field. As a result, meta-analyses may not adequately capture the comprehensive and multifaceted landscape of basic, translational, and clinical research within the TME. Conversely, bibliometric analysis offers a macro-level perspective that elucidates the dynamic evolution and knowledge structure of research through quantitative indicators, such as publication counts, citation frequencies, and co-citation rates. These metrics facilitate the comprehensive evaluation of research trends, the identification of collaborative networks, and the quantification of scientific contributions. Furthermore, bibliometrics employs visualization techniques to effectively display research hotspots and emerging fields, a capability that traditional narrative reviews and meta-analyses lack. This distinctive quantitative and visual capacity positions bibliometrics as a vital tool to complement and enhance the existing research on the TME of ovarian cancer.

In the domain of ovarian cancer research, some bibliometric analyses have been conducted across various sub-fields, including immunotherapy (18), ubiquitinase related to ovarian cancer (19), ovarian cancer stem cells (20), regulatory T cells in ovarian cancer (21), and biomarkers related to ovarian cancer drug resistance (22). These studies employ quantitative analysis to elucidate research trends, hotspots, and the impact within their respective subfields, thereby significantly contributing to advancements in ovarian cancer treatment. They not only synthesize existing research findings but also offer valuable insights for future investigations by pinpointing research hotspots and emerging directions. For instance, bibliometric studies focused on immunotherapy facilitate the allocation of resources towards comprehensive research, potentially expediting the application and refinement of immunotherapy in ovarian cancer (18). Similarly, investigations into drug resistance biomarkers provide novel perspectives for exploring biomarkers associated with drug resistance (22). While bibliometric analyses have enriched different aspects of ovarian cancer research, there remains a lack of bibliometric analyses focused specifically on the TME of ovarian cancer. This study applies bibliometric and visual analysis methodologies to elucidate research trends and focal areas within TME associated with ovarian cancer. Despite advancements, the treatment of ovarian cancer continues to encounter significant challenges, with the TME being pivotal in the pathogenesis, progression, immunosuppression, and inflammatory responses of the disease. A comprehensive review of the current research landscape concerning the ovarian cancer microenvironment can be achieved through systematic metrological and visual analyses of pertinent literature. For instance, CiteSpace and VOSviewer software are employed to construct a knowledge map of this research domain, facilitating a visual analysis of the number of publications, authors, institutions, and keywords (23). We expect to provide directions and important reference information for the in-depth research in this field.

## 2 Materials and methods

### 2.1 Data collection and search strategies

**Figure 1:**
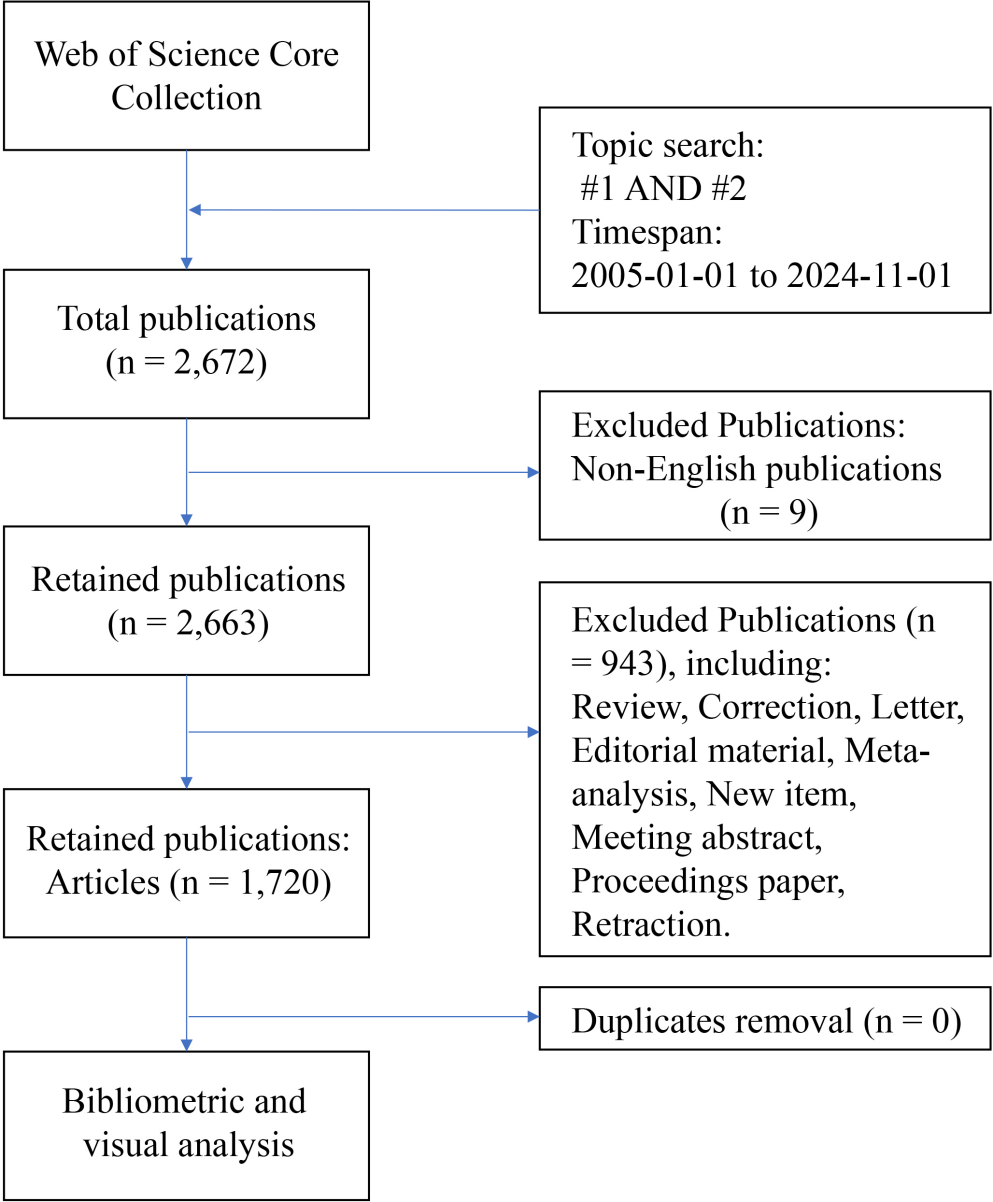
The flow chart of literature retrieval of the study.

The data used in this study were obtained from the Web of Science Core Collection (WOSCC) database. The literature retrieval formula of this study: TS = (“ovarian cancer*” OR “ovarian carcinoma*” OR “ovarian neoplasm*” OR “ovary carcinoma*” OR “ovary cancer*” OR “ovary neoplasm”); #2: TS = (“tumor microenvironment*” OR “cancer microenvironment*” OR “cellular microenvironment*” OR “cell microenvironment*”); #3: #1 AND #2. The inclusion criteria were as follows: a) Timespan: from January 1st, 2005, to November 1st, 2024; b) Language: English; and c) Publication type: article. The exclusion criteria were as follows: a) documents with irrelevant topics (such as only referring to ovarian cancer but not mentioning microenvironment, or only studying microenvironment but not related to ovarian cancer); B) other types of publications except articles. The literature retrieval and screening process was conducted independently by two authors. Any discrepancies were resolved through consultation with a third author. Following analysis with CiteSpace software, no duplicate documents were identified, resulting in a final selection of 1,720 articles. The literature retrieval process is illustrated in **Figure 1**.

### 2.2 Data analysis and visualization

CiteSpace and VOSviewer are the most widely adopted tools for bibliometric analysis and visual analysis. CiteSpace excels at extracting and analyzing latent information from the literature, whereas VOSviewer offers advanced visualization capabilities (24, 25).

## 3 Results

### 3.1 Publication trend analysis

**Figure 2:**
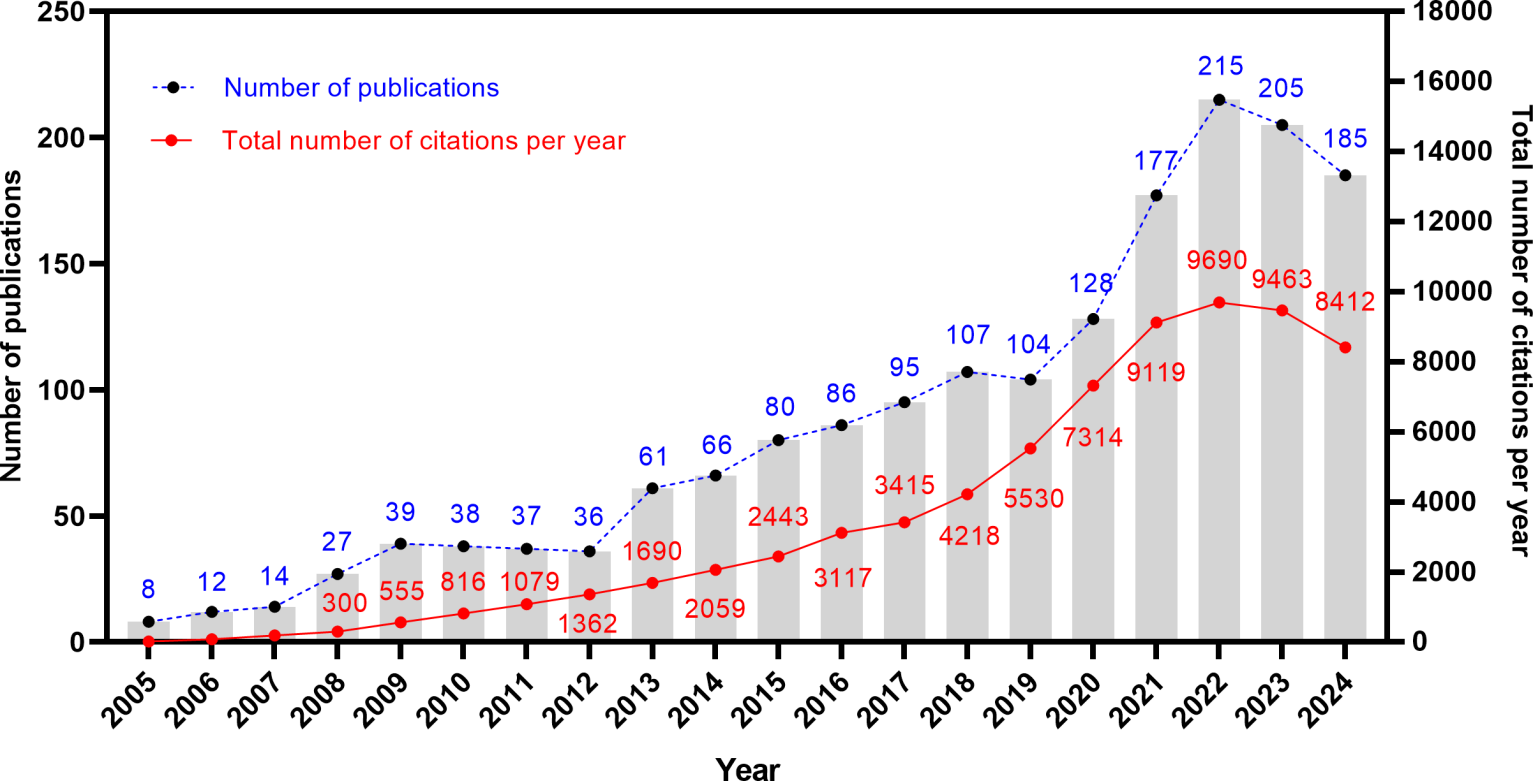
Trends in publication output and citation frequency about the field.

A total of 1,720 articles related to the TME of ovarian cancer were obtained. **Figure 2** shows the publication trajectory from 2005 to 2024, which was divided into four distinct phases with a sustained upward trend. From 2005 to 2011, the growth in publications was relatively modest, with 175 articles published, representing 10.2% of the total. From 2012 to 2018, there was a steady increase in the number of publications, with 531 articles published, constituting 30.9% of the total. The period from 2019 to 2022 witnessed a marked acceleration in the growth rate, culminating in 624 publications, which accounted for 36.3% of the total. During the 2023–2024 period, the publication volume remained at a high level. The publication trend, as depicted by the fitting curve (y = 10.57 * x–21204, R^2^ = 0.9037, *p* ≤ 0.0001) in **Supplementary Figure S1**, indicates a strong linear correlation between the year and the number of publications. As of now, data for only the first ten months of 2024 have been collected. Based on the observed publishing trend and the fitting curve, it is reasonable to predict a continued increase in the number of publications in the future. Furthermore, the annual growth in the total number of citations underscores the escalating significance and attention being directed towards this research field.

### 3.2 Countries and institutions

Our bibliometric analysis reveals that numerous countries and scientific research institutions have significantly contributed to the advancement of this research domain. The United States led with 678 articles, followed by China with 599 articles. Other notable contributors included Germany (n=97), Italy (n=90), and Japan (n=74) (**Table 1**). Furthermore, countries exhibiting the highest betweenness centrality scores were Belgium (0.54), Spain (0.4), and Japan (0.36), indicating their pivotal role as intermediaries within this research field. The term “centrality” refers to the degree of a node’s (such as a country, institution, or author) importance within a network, reflecting its capacity to act as a bridge or intermediary between different parts of the network. A higher centrality value indicates that the node plays a more significant role in disseminating information and knowledge.

**Table 1:** The top 15 countries and institutions about the field.

Rank	Country	Counts	Centrality	Institution	Counts	Centrality
1	USA	678	0.11	University of Texas System	85	0.08
2	CHINA	599	0.11	University of California System	54	0.03
3	GERMANY	97	0.2	Harvard University	53	0.18
4	ITALY	90	0.17	Institut National de la Sante et de la Recherche Medicale (Inserm)	48	0.14
5	JAPAN	74	0.36	Fudan University	46	0.02
6	CANADA	69	0.06	Shanghai Jiao Tong University	43	0.05
7	UK	64	0.25	Roswell Park Comprehensive Cancer Center	39	0.09
8	FRANCE	61	0.24	Memorial Sloan Kettering Cancer Center	32	0.12
9	SOUTH KOREA	56	0.13	Sun Yat Sen University	30	0.01
10	POLAND	44	0.29	Cornell University	30	0.03
11	AUSTRALIA	43	0.09	National Institutes of Health (NIH) - USA	28	0.16
12	INDIA	33	0.11	Nanjing Medical University	27	0.02
13	NETHERLANDS	30	0.07	Johns Hopkins University	25	0.1
14	SWEDEN	23	0.17	Pennsylvania Commonwealth System of Higher Education (PCSHE)	25	0.03
15	SPAIN	22	0.4	Chinese Academy of Sciences	24	0

**Figure 3:**
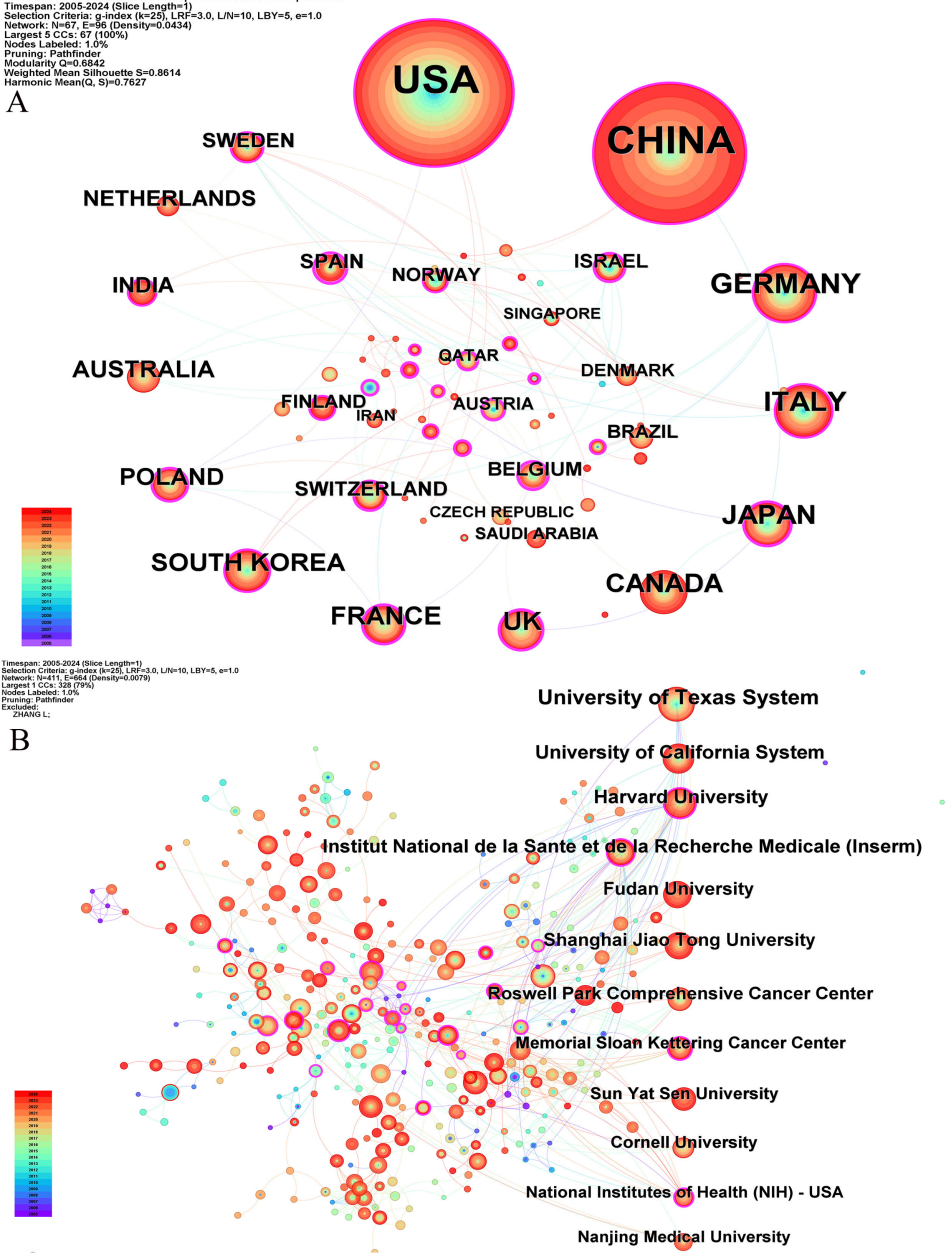
The co-occurrence map of countries **(A)** and institutions **(B)** about the research field. The number of publications is arranged clockwise from large to small.

A single publication may encompass multiple institutional affiliations, including subsidiaries within the same overarching organization. Upon verification of the information and data through the WoSCC database, we manually consolidated different departments associated with the same organization. Specifically, if there are multi-level institutional signatures, they will be unified into the name of the highest-level institution. At the institutional level, the University of Texas System achieved the highest ranking with 85 published articles, followed by the UTMD Anderson Cancer Center with 75 articles, and the University of California System with 54 articles (**Table 1**). The substantial volume of publications from these institutions indicates a significant investment of research resources in this domain, reflecting a high level of research activity. **Figure 3** illustrates that recent years have witnessed extensive collaboration among countries and institutions, which has accelerated scientific progress in the field of ovarian cancer TME.

### 3.3 Journals

**Figure 4:**
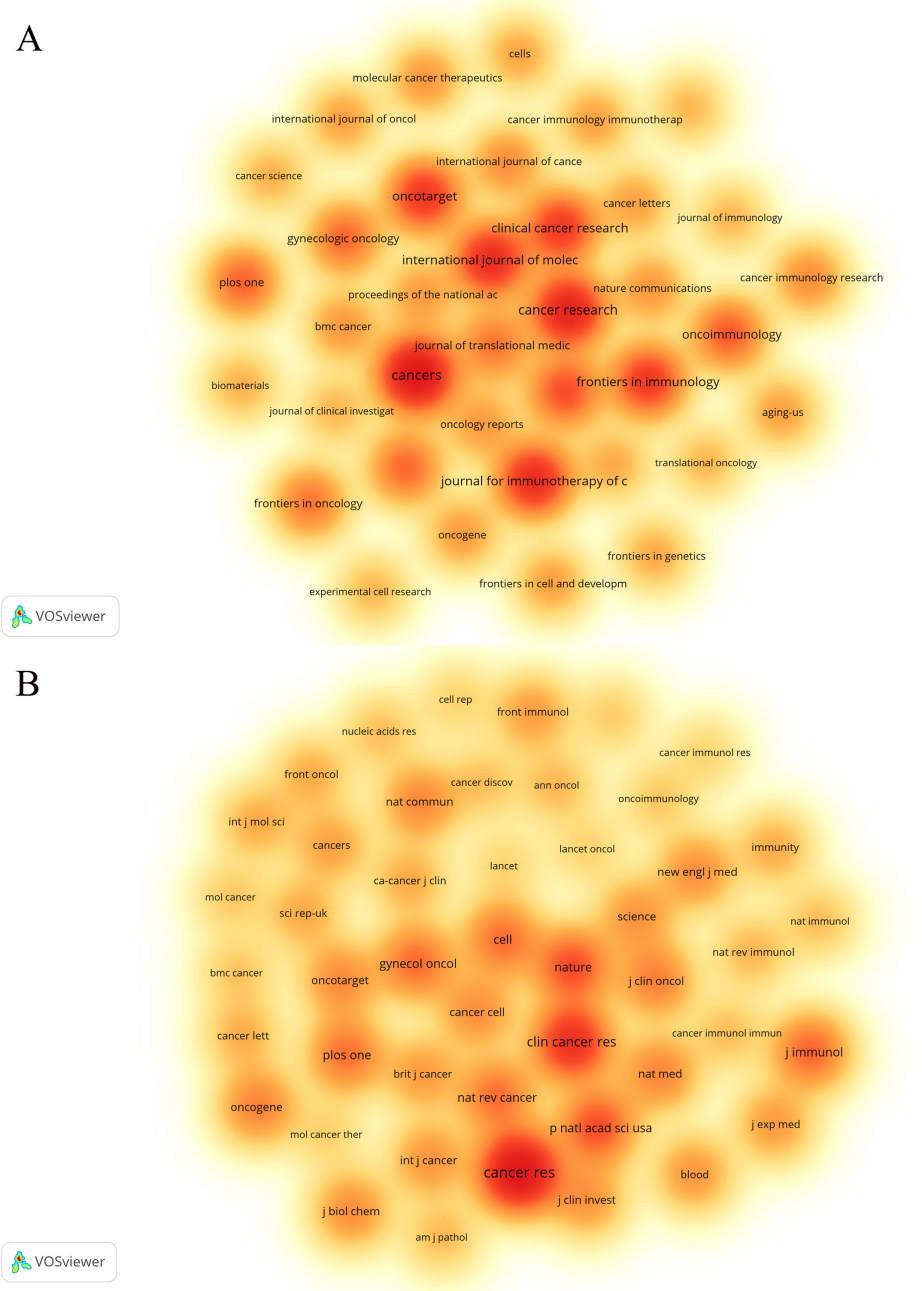
The density map of journals **(A)** and co-cited journals **(B)** about the field. **Figure 3A** shows journals with a number of publications ≥10; **Figure 3B** shows the journals with citations ≥350.

Statistical analysis revealed that a total of 1,720 articles were disseminated across 453 academic journals. Notably, the leading journals in terms of publication volume included *Cancers* (n=67), *Cancer Research* (n=53), and the *Journal for Immunotherapy of Cancer* (n=47). **Figure 4A** shows these high-volume journals. These journals have become one of the main publishing platforms for achievements in this research field. Utilizing statistical analysis of citation frequencies, we identified several highly cited journals. Notably, *Cancer Research* emerged as the most frequently cited journal, amassing a total of 6,812 citations, followed by *Clinical Cancer Research*, which garnered 3,837 citations (refer to **Table 2**). Furthermore, *Cancer Research* (n=3,966) and *Clinical Cancer Research* (n=2,649) were also the most frequently co-cited journals (refer to **Table 2**, **Figure 4B**). These findings indicate that both journals exert substantial influence in this field.

**Table 2:** The top 10 journals and co-cited journals.

Rank	Journal	Citations	Counts	Co-cited journal	Co-Citations
1	Cancer Research	6812	53	Cancer Research	3966
2	Clinical Cancer Research	3837	39	Clinical Cancer Research	2649
3	Proceedings of the National Academy of Sciences of the United States of America	3276	15	Nature	1887
4	Journal of Experimental Medicine	2087	4	Proceedings of the National Academy of Sciences of the United States of America	1867
5	Oncotarget	1866	41	Journal of Immunology	1575
6	Journal of Clinical Investigation	1674	10	Gynecologic Oncology	1491
7	Oncoimmunology	1498	29	Cell	1437
8	Nature Communications	1486	16	PLoS One	1429
9	Journal for Immunotherapy of Cancer	1260	47	Nature Reviews Cancer	1424
10	Blood	1226	4	Journal of Clinical Oncology	1231

### 3.4 Authors

**Figure 5:**
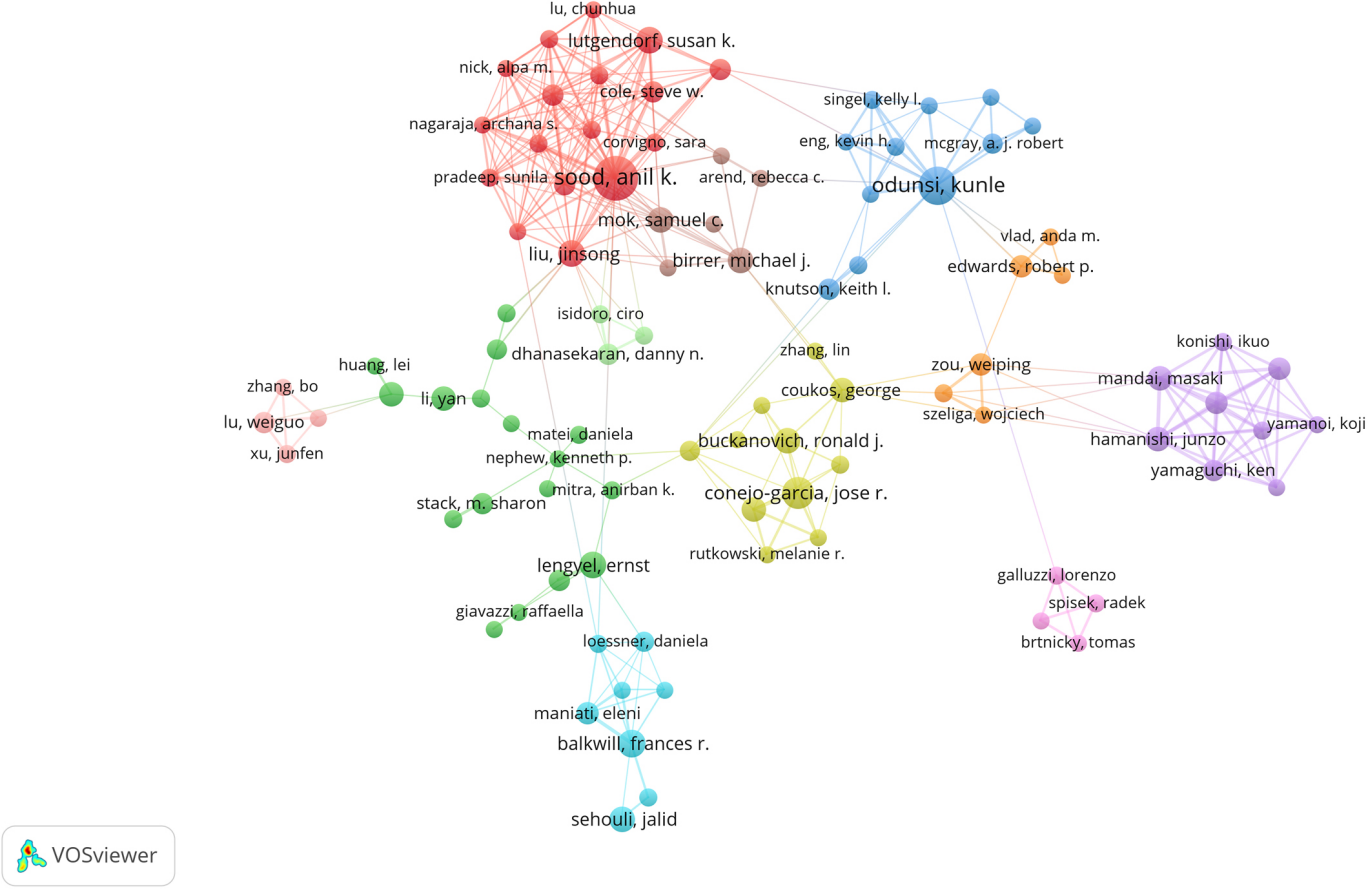
The visualization map of authors about the field. Minimum number of documents of an author ≥ 5.

A total of 11,892 authors contributed to the co-authorship of these articles. **Figure 5** illustrates the collaborative network of these authors along with their respective publication counts. Notably, Anil K. Sood, with 34 publications, emerged as the most prolific researcher in this field, followed by Kunle Odunsi with 25 publications, and Jose R. Conejo-Garcia with 18 publications. Furthermore, the collaborative network diagram revealed the presence of approximately 10 major research groups within this domain. An analysis of cited authors can provide insights into scholars with significant influence in a particular field. As shown in **Table 3**, the most frequently cited researcher is Weiping Zou (n=2,553), followed by Anil K Sood (n=2,458) and Ilona Kryczek (n=2,008).

**Table 3:** The top 10 cited authors.

Rank	Cited author	Citations	Counts
1	Weiping Zou	2553	9
2	Anil K Sood	2458	34
3	Ilona Kryczek	2008	6
4	Wojciech Szeliga	1943	5
5	Thorsten Hagemann	1643	4
6	Frances R Balkwill	1547	13
7	Shuang Wei	1430	4
8	Junzo Hamanishi	1400	10
9	Masaki Mandai	1400	10
10	Linda Vatan	1321	4

### 3.5 The keyword analysis

**Figure 6:**
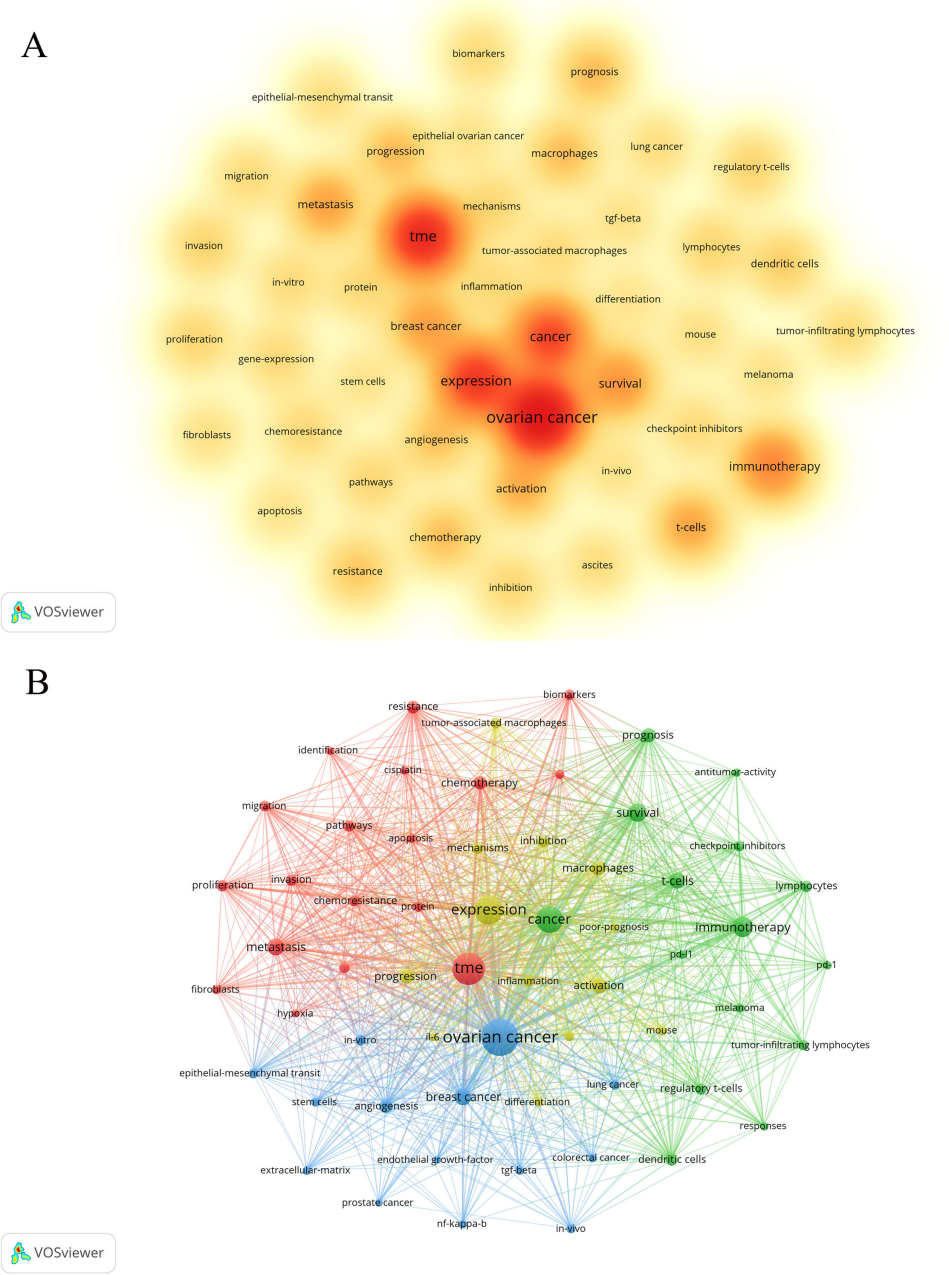
The co-occurrence density map **(A)** and network **(B)** of keywords about the field. Minimum number of occurrences of keywords ≥50. Minimum number of occurrences of keywords ≥40.

Keywords, as the core of literature content, can reflect the focus of research. Following the cleaning and consolidation of the initial keyword dataset, a total of 6,086 distinct keywords were identified. **Table 4** presents the top 20 keywords, with “ovarian cancer” (n=1028) being the most frequently occurring, followed by “TME” (n=628) and “expression” (n=505). **Figure 6A** visually summarizes the high-frequency keywords. By integrating these frequently occurring keywords, we can preliminarily identify key research areas within this field, including the functional analysis of various cells within the TME of ovarian cancer, investigations into the TME’s role in ovarian cancer invasion, metastasis, and prognosis, as well as studies on TME-related drug resistance and the mechanisms of angiogenesis. Include keywords that appear at least 40 times (**Figure 6B**). Through the visual keyword co-occurrence network, we can intuitively observe the relationship and closeness between keywords. After cluster analysis, four clusters were obtained, which may represent four main research areas. The representative keyword of cluster 1 (red) is “TME”. The representative keyword of Cluster 2 (green) is “cancer”. The representative keyword of Cluster 3 (blue) is “ovarian cancer”. The representative keyword of Cluster 4 (yellow) is “expression”. These four keyword clusters respectively represent four different research directions/scopes, including 1) The TME and the biological processes of ovarian cancer cells, including proliferation, migration, invasion, metastasis, and mechanisms of drug resistance; 2) Investigations into the immunotherapy of ovarian cancer, with a particular focus on the impact of the TME on immune checkpoint inhibitors (ICIs); 3) Exploring the mechanism of the TME and angiogenesis or epithelial-mesenchymal transition (EMT); and 4) The study on inflammation and TAMs in the ovarian cancer TME.

**Table 4:** Top 20 keywords related to the research field.

Rank	Keyword	Counts	Rank	Keyword	Count
1	ovarian cancer	859	11	prognosis	122
2	TME	628	12	progression	122
3	expression	505	13	macrophages	119
4	cancer	436	14	chemotherapy	116
5	immunotherapy	256	15	angiogenesis	109
6	survival	210	16	resistance	102
7	breast cancer	179	17	dendritic cells	93
8	metastasis	176	18	invasion	84
9	t-cells	168	19	regulatory t-cells	84
10	activation	147	20	proliferation	82

TME, tumor microenvironment.

**Figure 7:**
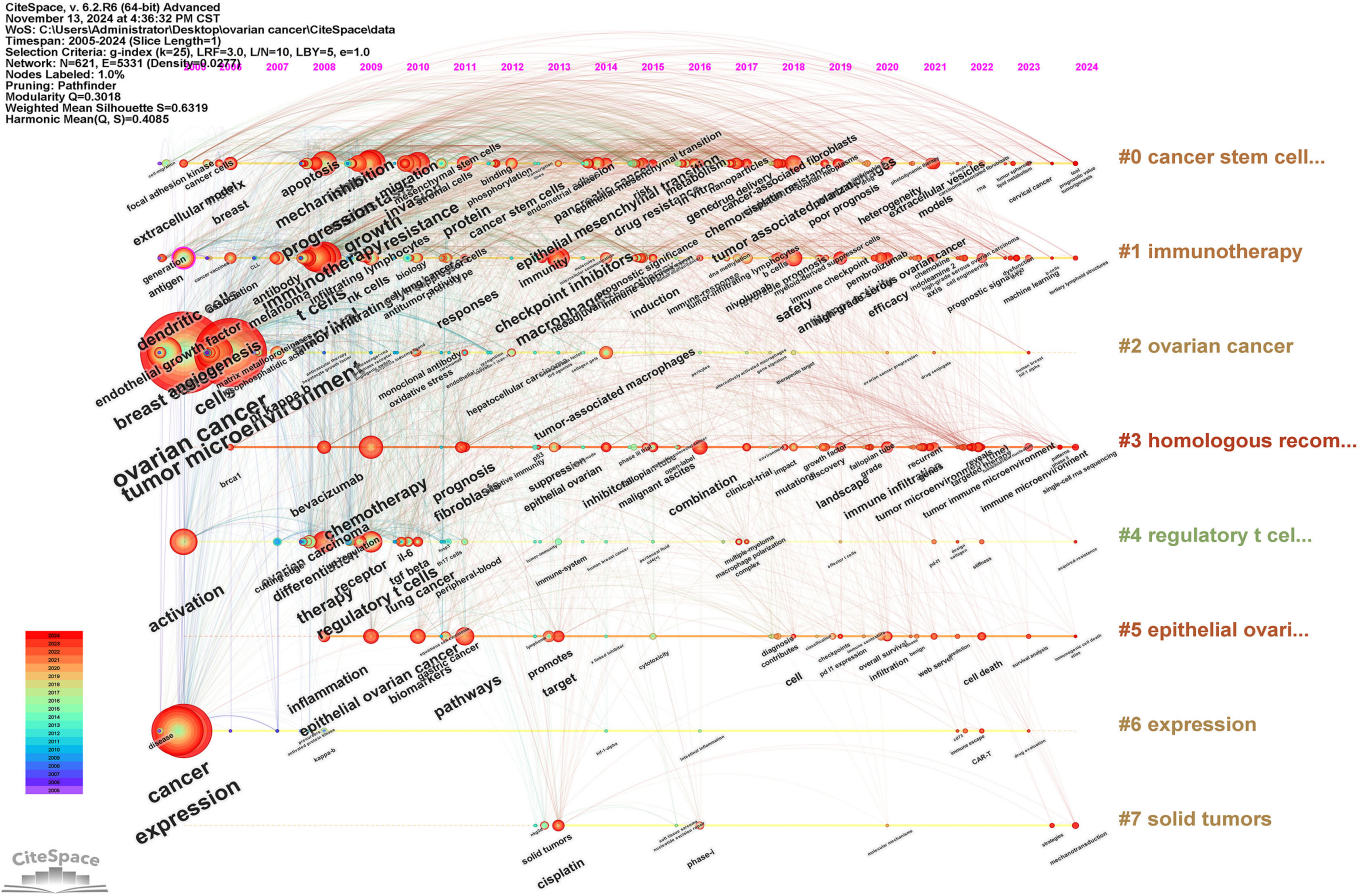
The timeline viewer of keywords about the field. The circle represents a keyword and the horizontal line represents a cluster.

The keyword timeline viewer can help researchers better understand the development of research fields and identify hot topics, and provide guidance for future research. **Figure 7** illustrates that the distribution of keywords varies across different years. In certain years, keywords appear relatively dense, suggesting that research outcomes were prolific and encompassed a broad range of topics during these periods. Conversely, in other years, the keywords are comparatively sparse, potentially indicating that the research was in a stable or transitional phase. A more detailed analysis of these trends will be provided in the discussion section.

### 3.6 Co-cited articles and reference burst

The co-citation analysis holds substantial importance for identifying the key research achievements and hot research topics in this field. By conducting a co-citation analysis of literature about the field, we identified several highly co-cited documents (refer to **Table 5**) (26–35). The publication dates of these documents predominantly span from 2015 to 2020. Among them, the clinical trial study “Anti-tumor activity and safety of Pembrolizumab in patients with advanced recurrent cancer: results from the phase II KEYNOTE-100 study (26) “ published by Matulonis et al. in 2019 was co-cited the most times, with a total of 61 times.

**Table 5:** The top 10 co-cited articles related to the research field.

Rank	Author	Title	Journal	Year	Co-citations	Centrality
1	Matulonis et al. (26)	Antitumor activity and safety of pembrolizumab in patients with advanced recurrent ovarian cancer: results from the phase II KEYNOTE-100 study	Annals of Oncology	2019	61	0.03
2	Izar et al. (27)	A single-cell landscape of high-grade serous ovarian cancer	Nature Medicine	2020	37	0.03
3	Hamanishi et al. (28)	Safety and Antitumor Activity of Anti-PD-1 Antibody, Nivolumab, in Patients with Platinum-Resistant Ovarian Cancer	Journal of Clinical Oncology	2015	35	0.20
4	Hornburg et al. (29)	Single-cell dissection of cellular components and interactions shaping the tumor immune phenotypes in ovarian cancer	Cancer Cell	2021	27	0.02
5	Mariathasan et al. (30)	TGFβ attenuates tumour response to PD-L1 blockade by contributing to exclusion of T cells	Nature	2018	25	0.02
6	Ray-Coquard et al. (31)	Olaparib plus Bevacizumab as First-Line Maintenance in Ovarian Cancer	New England Journal of Medicine	2019	22	0.02
7	Disis et al. (32)	Efficacy and Safety of Avelumab for Patients with Recurrent or Refractory Ovarian Cancer: Phase 1b Results from the JAVELIN Solid Tumor Trial	JAMA Oncology	2019	22	0.10
8	Pearce et al. (33)	Deconstruction of a Metastatic Tumor Microenvironment Reveals a Common Matrix Response in Human Cancers	Cancer Discovery	2018	22	0.04
9	González-Martín et al. (34)	Niraparib in Patients with Newly Diagnosed Advanced Ovarian Cancer	New England Journal of Medicine	2019	22	0.01
10	Jiménez-Sánchez et al. (35)	Unraveling tumor-immune heterogeneity in advanced ovarian cancer uncovers immunogenic effect of chemotherapy	Nature Genetics	2020	21	0

**Figure 8:**
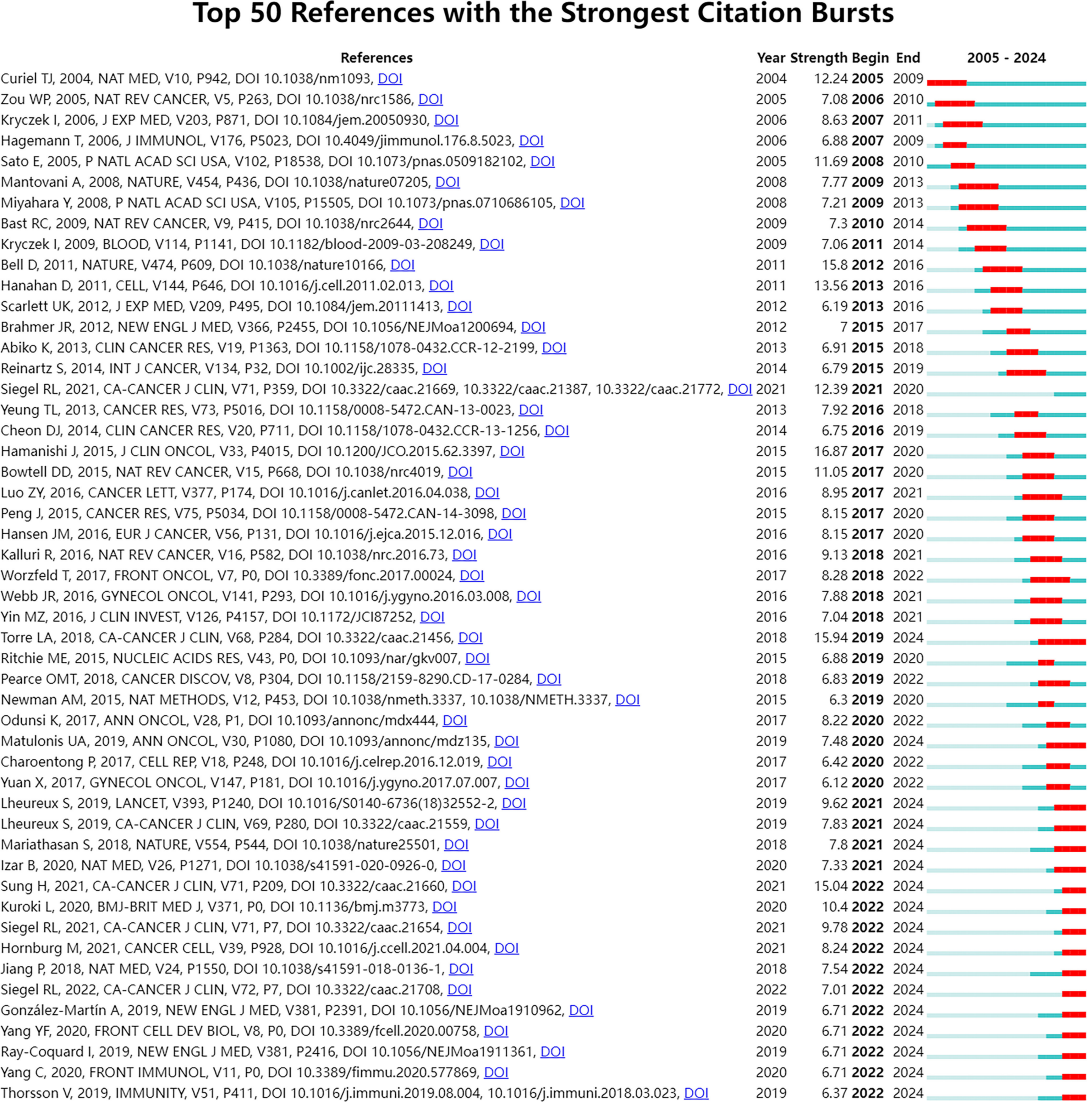
Top 50 references with the strongest citation bursts. The Blue bars mean the reference had been published; the red bars mean citation burst.

To further investigate the emerging topics within this research domain, we conducted a sudden citation analysis of the relevant literature. “Citation burst” is a phenomenon where a particular study or keyword experiences a sudden and significant increase in the number of citations within a relatively short period. This usually indicates that the research topic or finding has garnered considerable attention and is likely at the forefront of the field. **Figure 8** presents the top 50 references with the strongest citation bursts. Notably, 17 of these articles are in a state of citation bursts, constituting 34% of the total. Furthermore, these 17 articles were published between 2018 and 2022, indicating that they have been frequently cited in recent years.

## 4 Discussion

Ovarian cancer remains a major challenge in the field of oncology and one of the main causes of death in gynecological malignant tumors. The TME has emerged as a crucial factor in ovarian cancer. It is composed of a complex array of cellular and non-cellular components that interact with tumor cells. The TME is instrumental in the carcinogenesis, progression, and metastasis of ovarian cancer, and it is closely linked to prognosis and therapeutic response (7, 36). Consequently, further comprehensive investigation of the ovarian cancer TME is imperative.

### 4.1 General information

The sustained rise in publications about this research domain represents a significant trend. This upward trajectory can be ascribed to several factors. Primarily, the heightened awareness of the critical role of the TME in the pathogenesis of ovarian cancer has piqued the interest of researchers. With more studies highlighting its role in tumor growth, metastasis, and drug resistance, scientists are increasingly inclined to explore this field further. Secondly, advancements in technology have furnished researchers with more advanced tools for investigating the TME at the molecular and cellular levels, exemplified by single-cell sequencing technology (37, 38). These tools facilitate more comprehensive and detailed research. Thirdly, the intricate nature of the TME, encompassing immune cells, fibroblasts, and the extracellular matrix, provides extensive research opportunities, particularly in understanding the interactions among these components and their roles in the progression and metastasis of ovarian cancer (39–41). In addition, the influence of TME on the recruitment and function of immune cells and its role in creating an immunosuppressive environment further emphasize the importance of studying this microenvironment to improve the treatment results and the prognosis of patients.

We have observed that the United States and China occupy a leading position in the field of the ovarian cancer TME, which may reflect the strong strength and resource investment of these two countries in the field of biomedical research. The University of Texas System has produced the most research results in this field, which shows that the institution has remarkable scientific research ability and concentration in ovarian cancer research. In addition, 70% of the top 10 research institutions are from the United States, which further highlights the leading position of the United States in this research field. These results reveal the global geographical distribution of the ovarian cancer TME research and the concentrated distribution of research productivity, and also highlight the importance of international cooperation in promoting progress in this field. The centrality score serves as a crucial metric in bibliometric analysis, employed to assess the extent to which a node—such as a country or institution—functions as a “bridge” or “intermediary” within the research network. A high centrality score (e.g., Belgium: 0.54) signifies that the entity is instrumental in facilitating collaboration and the exchange of knowledge in the domain of the ovarian cancer microenvironment. Belgium, Spain, and Japan publish less than the United States and China. Nevertheless, their high centrality scores mark them as key intermediaries and collaborative hubs in global ovarian cancer microenvironment research. This underscores the critical importance of international collaboration, as these hubs are vital in integrating diverse expertise and expediting scientific advancement. Furthermore, countries with high centrality scores may play a pivotal role in resource integration, research collaboration, and academic leadership. Weiping Zou, Anil K Sood, and Ilona Kryczek are the most cited researchers. *Cancer Research* and *Clinical Cancer Research* are journals that are frequently cited. This may mean that their research has an important influence and recognition in academic circles, and their work may represent the research hotspot in this field.

### 4.2 Research hotspot analysis—based on keywords and co-cited documents

In the domain of the ovarian cancer TME, the analysis based on keywords and co-cited documents has become an important means to reveal the research hotspots and their dynamic changes. Through this method, we can dig deep into the key information hidden behind many research results, so as to grasp the core research direction in this field. Through the clustering analysis algorithm, the keywords in the field of the ovarian cancer TME are divided into four clusters (**Figure 6B**). Each cluster probably represents the related research direction in this research field. The four research directions encompass:

1) The TME and the biological processes of ovarian cancer cells, including proliferation, migration, invasion, metastasis, and mechanisms of drug resistance;

The TME plays a crucial role in the pathophysiology of ovarian cancer. The high mortality rate associated with ovarian cancer is primarily due to its propensity for peritoneal metastasis and the formation of drug resistance (42). The TME comprises a diverse array of cell types and molecules that interact with cancer cells via intricate signal transduction networks, thereby facilitating the proliferation, migration, invasion, and metastasis of cancer cells (43). Within the TME of ovarian cancer, cancer-associated fibroblasts (CAFs) serve as key regulators, promoting tumor invasion and metastasis through the secretion of various cytokines and growth factors (44). Drug resistance has emerged as a major obstacle in ovarian cancer therapy. The TME promotes this resistance through several distinct mechanisms. Research indicates that ovarian cancer cells can transfer drug resistance genes to other cells through exosomes, thus enhancing the overall drug resistance (42). Furthermore, the interaction between the extracellular matrix (ECM) and cells within the TME is crucial in the development of drug resistance (45). A comprehensive investigation into the mechanisms governing the interaction between the TME and ovarian cancer cells is anticipated to facilitate the development of more effective therapeutic strategies, thereby enhancing the survival rates of patients with ovarian cancer.

2) Investigations into the immunotherapy of ovarian cancer, with a particular focus on the impact of the TME on ICIs;

The TME of ovarian cancer comprises cancer cells, immune cells, fibroblasts, and various extracellular matrix components. These constituents collectively contribute to the establishment of an immunosuppressive milieu, thereby impeding the efficacy of ICIs (46). Notably, tumor-associated macrophages (TAMs), regulatory T cells (Tregs), and myeloid-derived suppressor cells (MDSCs) are enriched in the TME of ovarian cancer, further exacerbating immunosuppression (47). Moreover, the immune evasion mechanisms inherent to ovarian cancer significantly impact the success of immunotherapeutic interventions. Research indicates that the low density of tumor-infiltrating lymphocytes (TILs) in ovarian cancer, along with both cellular and non-cellular interactions within the microenvironment, are critical factors contributing to the resistance against ICIs (48). Consequently, elucidating the immune microenvironment and its molecular underpinnings in ovarian cancer holds substantial clinical importance for enhancing the efficacy of immunotherapy (49).

In the investigation of the TME in ovarian cancer, research on TILs and TAMs has increasingly emerged as a significant area of interest. These immune cells are pivotal in the initiation, progression, and therapeutic response of ovarian cancer. Studies on the TME in early-stage ovarian cancer have primarily concentrated on the interactions between tumor cells and the extracellular matrix, blood vessels, and other structural components, with comparatively less emphasis on immune cells. However, advancements in immunology have highlighted the critical role of immune cells in tumorigenesis and tumor progression. Consequently, TILs and TAMs, as key immune cell types, have garnered attention and initiated preliminary explorations in this research domain. Subsequent investigations revealed that immune cells within the TME of ovarian cancer exhibit aberrant functionality. For instance, TILs encounter challenges in maintaining their prolonged anti-tumor activity due to the presence of immunosuppressive molecules secreted by tumor cells and the inhibitory influence of regulatory immune cells (50). Furthermore, TAMs are polarized towards the immunosuppressive M2 phenotype, thereby facilitating tumor progression (51). In this context, research efforts began to concentrate on elucidating the dysfunction and underlying mechanisms of TILs and TAMs. Following the advent of immunotherapy, the significance of TILs and TAMs in research has been further accentuated. Immunotherapeutic approaches, such as immune checkpoint inhibitors, exhibit limited efficacy in treating ovarian cancer, a limitation closely associated with the status of TILs and TAMs (48). Consequently, research efforts have increasingly focused on elucidating the mechanisms by which TILs and TAMs influence the efficacy of immunotherapy. Concurrently, strategies for combined immunotherapy targeting TILs and TAMs have been proposed, leading to a surge in related research endeavors. In recent years, advanced techniques such as single-cell sequencing have significantly contributed to the comprehensive investigation of TILs and TAMs. These technologies facilitate precise analysis of the heterogeneity, subgroup composition, and intercellular interactions of TILs and TAMs, thereby elucidating their intricate mechanisms of action within the TME of ovarian cancer. Consequently, this further promotes the related research to become a field of great concern.

3) Exploring the mechanism of the TME and angiogenesis or epithelial-mesenchymal transition (EMT);

EMT involves the conversion of epithelial cells into mesenchymal cells, a process characterized by substantial alterations in cell morphology and gene expression. This transformation facilitates the ability of cancer cells to penetrate the basement membrane and invade adjacent tissues (52). In the context of ovarian cancer, EMT not only enhances tumor invasion and metastasis but is also intricately linked to chemotherapy resistance (53). Moreover, angiogenesis represents another critical factor in the progression and metastasis of ovarian cancer. Through the provision of essential oxygen and nutrients, angiogenesis supports tumor growth and offers a conduit for cancer cell dissemination. Ovarian cancer cells have been shown to promote neovascularization by secreting angiogenic factors, such as vascular endothelial growth factor (VEGF) (54). Additionally, cytokines and signaling pathways associated with EMT, including the TGF-β and Wnt signaling pathways, also play a significant role in the angiogenic process (55, 56). The TME of ovarian cancer regulates EMT and angiogenesis via intricate intercellular interactions and signal transduction pathways. The in-depth study of these mechanisms not only helps us understand the invasion and metastasis mechanisms of ovarian cancer but also provides potential targets for developing new therapeutic strategies (57).

4) The study on inflammation and TAMs in the ovarian cancer TME.

The involvement of TAMs in ovarian cancer has been extensively investigated. TAMs show unique activation characteristics in ovarian cancer, which can create an immunosuppressive microenvironment, enable tumors to escape immune detection, and promote tumor progression (58). TAMs can directly enhance the invasive potential and chemotherapy resistance of ovarian cancer cells through the secretion of various cytokines, chemokines, enzymes, and exosomes (59). Moreover, the interaction between TAMs and other immune cells, such as lymphocytes, NK cells, and dendritic cells, suppresses their activity, thereby leading to immunosuppression (59). Within the TME of ovarian cancer, the polarization state of TAMs significantly influences tumor prognosis. A high M1/M2 ratio has been associated with improved overall survival (OS) and progression-free survival (PFS) (60). Furthermore, IL-10 secreted by TAMs can enhance the proliferation and function of Tregs, thereby facilitating tumor progression (61). Consequently, therapeutic strategies aimed at targeting TAMs, including the inhibition of their immunosuppressive functions or the reprogramming of their polarization states, may offer novel avenues and insights for advancing cancer immunotherapy (62).

The co-citation analysis can help identify highly cited papers that have an important influence in a specific field. The greater the co-citation intensity, the more similar they are in content, which is helpful to find similar research topics in the research field (63). In this study, the top 10 co-cited articles (26–35) were selected (**Table 5**). We summarized and concluded these articles. Three studies explored the anti-tumor activity and safety of ICIs such as Pembrolizumab, Nivolumab, and Avelumab in the treatment of ovarian cancer (26, 28, 32). These studies showed that, although immune checkpoint blocking therapy was effective in ovarian cancer, the objective response rate (ORR) was relatively low, and there were differences in response related to the expression level of PD-L1. This suggests that immune checkpoint blocking therapy is an important research direction in the treatment of ovarian cancer. However, it is necessary to further explore how to improve the curative effect and predict which patients are most likely to respond to treatment. Two studies (27, 29) analyzed the variability of cell composition and functional programs in the TME of ovarian cancer by single-cell RNA sequencing (scRNA-seq) technology, and revealed the key determinants and biological basis of tumor immunophenotype. These studies were helpful to understand the complexity and dynamics of the TME and provide important information for developing new treatment strategies. One study (30) explored the role of TGFβ signal in the TME, especially in limiting T cell infiltration, which may affect the response to PD-L1 blocking therapy. This indicates that the TGFβ signaling pathway may be a potential therapeutic target and can be used to improve the immunotherapy response of ovarian cancer. Two studies (31, 34) explored the role of PARP inhibitors such as Olaparib and Niraparib in the treatment of ovarian cancer, especially in patients with BRCA mutation and homologous recombination defect (HRD). These studies showed that PARP inhibitors could not only directly inhibit the DNA repair of tumor cells but also enhance the therapeutic effect by affecting the TME. One study (33) described the evolving human metastatic microenvironment by analyzing the same high-grade serous ovarian cancer (HGSOC) for the first time, and revealed the complexity and dynamics of extracellular matrix remodeling in the process of metastasis. Furthermore, it suggested that certain human malignant tumors may exhibit common and potentially targetable matrix responses, which could influence the progression of the disease (33). One study (35) revealed that the tumor immune microenvironment (TIME) of advanced HGSOC was inherently heterogeneous through the analysis of immunogenomics, and chemotherapy could induce local immune activation, which indicated that chemotherapy could enhance the immunogenicity of immune-excluded HGSOC.

Following an extensive review of the literature, the primary research focal points within the domain of the TME in ovarian cancer have been identified as follows:

1. The utilization of immune checkpoint inhibitors, including Pembrolizumab, Nivolumab, and Avelumab, in the treatment of ovarian cancer.
2. The application of single-cell RNA sequencing technology in the investigation of the TME.
3. The TME heterogeneity.
4. The influence of TGF-β signaling and other pathways within the TME.
5. The impact of PARP inhibitors on the TIME.

In the domain of ovarian cancer microenvironment research, ICIs have led to significant advancements, exemplified by anti-PD-1/PD-L1 antibodies that target immune cells within the TME (64, 65). The TME is characterized by immunosuppression, where the function of immune cells, such as T cells, is compromised, hindering their ability to effectively eliminate tumor cells. By obstructing the associated pathways, ICIs facilitate the reactivation of T cells, thereby restoring their surveillance and cytotoxic capabilities against tumor cells. ICIs can also change the composition of the TME by reducing the proportion of immunosuppressed cells and increasing the infiltration of effector T cells (66–68). Studies have demonstrated that ICIs facilitate the infiltration and functionality of CD8+ T cells through the inhibition of immune checkpoint molecules, including PD-1 and CTLA-4, thereby ameliorating the TME (66). Within the TME, immunosuppressive cells, such as Tregs and TAMs, are pivotal in immunotherapy due to their role in suppressing the activity of effector T cells (67). Moreover, the infiltration of effector T cells can be further enhanced and the therapeutic outcome improved by combining ICIs with other treatments, such as radiotherapy or chemotherapy (69, 70). The recent surge in research on ICIs signifies a paradigm shift in the treatment of ovarian cancer, primarily driven by the inadequate efficacy of traditional therapies. The immunosuppressive nature of the ovarian cancer TME, characterized by elevated levels of PD-1/PD-L1 expression, provides a compelling biological rationale for targeting immune checkpoints (71). The increasing interest in ICIs has spurred extensive research into their clinical application for ovarian cancer treatment. During the clinical translation process, ICIs have evolved from monotherapy to the optimization of combination therapy strategies. Although the objective response rate (ORR) in ICIs monotherapy trials, such as KEYNOTE-100 (26), remains modest, these studies demonstrate the feasibility of ICIs as a monotherapy for ovarian cancer and underscore the necessity for predictive biomarkers and rational combination strategies. To address this challenge, researchers are actively investigating the integration of ICIs with other therapeutic modalities. While the use of ICIs in the treatment of ovarian cancer remains in its nascent stages, ongoing advancements in translational research are anticipated to yield more effective therapeutic strategies for patients with ovarian cancer in the future. Furthermore, our research highlights a significant interest in immunotherapy, particularly in ICIs and their integration with other therapeutic modalities. For instance, the combination of PD-1/PD-L1 inhibitors with PARP inhibitors has demonstrated enhanced therapeutic efficacy in the treatment of ovarian cancer (72, 73). The findings from our analysis not only illustrate the evolving research trends in this domain but also offer valuable insights for current and future clinical trials aimed at exploring these combinations.

Single-cell RNA sequencing (scRNA-seq) technology is a powerful analytical tool for exploring the field of the TME, and shows great potential and application value in TME research. Unlike conventional RNA sequencing, which offers an averaged gene expression profile across a tumor sample, scRNA-seq enables the elucidation of intercellular heterogeneity at the single-cell level. This capability facilitates a more profound comprehension of the heterogeneity and complexity inherent in the ovarian cancer TME (74). With this technique, researchers can accurately identify and differentiate various types of cells and characterize new cell subpopulations in the TME (75, 76). Furthermore, scRNA-seq plays an important role in the identification of novel biomarkers and therapeutic targets (77). For instance, Liu et al. (78) demonstrated the prognostic significance of M2-type TAMs infiltration in ovarian cancer by integrating scRNA-seq with large-scale bulk RNA sequencing. This study led to the identification of four novel biomarker genes associated with M2 TAMs: SLAMF7, GNAS, TBX2-AS1, and LYPD6. The expression levels of these genes are correlated with the survival prognosis of ovarian cancer patients, thereby offering new molecular markers for prognostic evaluation and therapeutic intervention in ovarian cancer. Gao et al. (79) identified the gene TNFRSF1B, which is closely associated with T-cell depletion, by analyzing the transcriptomic profile of depleted T cells, which may become a new immunotherapeutic target for ovarian cancer. scRNA-Seq technology provides a new perspective for the study of ovarian cancer TME, and lays the foundation for the development of personalized therapy and new therapy in the future.

The advent of scRNA-Seq technology has instigated transformative advancements in the investigation of the ovarian cancer microenvironment. Its core of promoting clinical transformation lies in the in-depth analysis of tumor cell heterogeneity. This technology enables an exhaustive examination of gene expression at the single-cell level, thereby elucidating the diversity and dynamic alterations of cell subsets within the TME. Such insights are instrumental for researchers seeking to comprehend the complexity of the TME in ovarian cancer and to identify novel therapeutic targets and biomarkers. In 2021, Hornburg et al. (29) conducted an in-depth analysis of cell composition, immune cell composition, and intercellular communication mechanisms across different modes of immune infiltration using single-cell analysis, thereby providing a novel theoretical foundation for the precision treatment of ovarian cancer. The utilization of scRNA-Seq technology facilitates a deeper understanding of the mechanisms underlying tumorigenesis, tumor progression, and drug resistance (80), thereby informing the development of novel therapeutic strategies. Furthermore, advancements in technology and decreasing costs have led to the increasingly widespread application of single-cell RNA sequencing in clinical research, offering substantial support for the translational research of ovarian cancer.

Ovarian cancer is a highly heterogeneous disease (81). The heterogeneity of the ovarian cancer TME is manifested in variations in cellular composition, cellular states, and cell-cell interactions both among different patients and within different regions of the same patient’s tumor. The TME of ovarian cancer plays a crucial role not only in tumor initiation and progression but also in influencing patient prognosis. Studies have shown that cell components in the TME, such as cancer-associated fibroblasts, immune cells, and endothelial cells, can interact through complex signal transduction pathways to promote tumor growth and metastasis (3). Moreover, modifications within the tumor TME can lead to phenotypic changes in tumor cells and promote stem-like properties, thereby complicating therapeutic interventions (82). In ovarian cancer, the heterogeneity of the TME is characterized by distinct immune cell infiltration patterns, which are significantly correlated with patient survival rates and treatment responses (83). For instance, a high infiltration of M1 macrophages is linked to a favorable prognosis in patients with high-grade and advanced ovarian cancer, whereas the presence of M2 macrophages is associated with a poorer prognosis (83). The TME heterogeneity provides a new idea for individualized treatment.

The TGFβ signaling pathway plays a pivotal and intricate role in the TME of ovarian cancer. It facilitates the invasion and metastasis of tumor cells by modulating extracellular matrix components and inducing epithelial-mesenchymal transition (EMT) (84, 85). Furthermore, the TGFβ signaling pathway is crucial in the immune evasion mechanisms of ovarian cancer. It suppresses the anti-tumor activity of immune cells, enabling tumor cells to evade immune surveillance and thereby advancing tumor progression. Neutralization of TGFβ has been shown to enhance tumor immunity and mitigate tumor progression in ovarian cancer patients (86). Additionally, the TGFβ signaling pathway is implicated in the regulation of tumor angiogenesis, thereby providing nutritional support essential for tumor growth (87).

In the TME of ovarian cancer, the effects of PARP inhibitors are multifaceted. PARP inhibitors affect the DNA repair machinery in tumor cells by inducing synthetic lethal effects, thus demonstrating significant therapeutic effects in BRCA 1/2-mutant cancers (88). The effects of PARP inhibitors are not limited to direct effects on cancer cells, they can also enhance antitumor effects by modulating immune responses in the TME (89). Research indicates that PARP inhibitors can facilitate the polarization of M1-type macrophages, enhance the antigen presentation capacity of dendritic cells, and promote the infiltration and cytotoxic activity of B cells and T cells (90). Furthermore, PARP inhibitors may inhibit immune cell activation and function by upregulating PD-L1 expression, potentially leading to immune evasion in certain contexts (89). This dual regulatory capacity endows PARP inhibitors with significant immunomodulatory potential in the treatment of ovarian cancer. Additionally, the integration of PARP inhibitors with other therapeutic strategies, such as ICIs, may enhance their effectiveness in the treatment of ovarian cancer (72). In a phase II clinical trial, Lampert et al. (72) evaluated the clinical efficacy and immunomodulatory effects of the PARP inhibitor Olaparib in conjunction with the anti-PD-L1 antibody Durvalumab for managing recurrent ovarian cancer. The findings indicated an overall response rate (ORR) of 14% and a disease control rate (DCR) of 71% among 35 patients. Additionally, the combination therapy demonstrated an immunomodulatory effect on the patients. By improving the TME, PARP inhibitors are able to improve the effectiveness of immunotherapy and overcome the tumor resistance to treatment (91). PARP inhibitors can overcome the adaptive resistance to PARP inhibitors by affecting immune cells in tumor microenvironment, such as TAMs (92). Furthermore, PARP inhibitors exert their effects not only through direct cytotoxicity on tumor cells but also by augmenting the anti-tumor immune response via stimulation of the stimulator of interferon genes (STING) pathway (92). Therefore, it is important to explore the mechanism of PARP inhibitors in TME for developing more effective treatment strategies.

The advancement of the clinical application of PARP inhibitors is attributed to their distinct anticancer mechanism. The PARP protein is integral to the DNA damage repair process within cells. By inhibiting PARP function, PARP inhibitors render DNA damage in tumor cells irreparable, thereby inducing apoptosis, particularly in tumor cells with homologous recombination repair deficiencies (93). In the context of ovarian cancer treatment, PARP inhibitors such as Olaparib, Niraparib, and Rucaparib have received approval for maintenance therapy in cases of platinum-sensitive recurrent ovarian cancer, demonstrating significant improvements in progression-free survival (PFS) during clinical trials (94). Furthermore, a study conducted by González-Martín et al. (34) assessed the efficacy of Niraparib in patients newly diagnosed with advanced ovarian cancer. The findings revealed a median PFS of 13.8 months for the Niraparib group compared to 8.2 months for the placebo group (hazard ratio, 0.62; 95% CI, 0.50 to 0.76; *p* < 0.001). The study provides critical data to support the optimization of clinical treatment strategies for ovarian cancer. As clinical trials have advanced, the efficacy and safety of PARP inhibitors have been substantiated, thereby facilitating their clinical application. Additionally, PARP inhibitors have the potential to enhance anti-tumor effects by modulating the TME, offering a novel perspective for clinical translation.

### 4.3 The evolution of hotspots and the exploration of emerging themes

The keyword timeline viewer (**Figure 7**) provides a comprehensive overview of the developmental trajectory of ovarian cancer TME research over the past decade. During the initial phase of TME research in ovarian cancer, spanning from 2005 to 2012, the primary focus was on elucidating the fundamental pathological characteristics of ovarian cancer, identifying the components of the TME, and exploring the application of conventional chemotherapy agents, such as cisplatin, alongside targeted therapies like bevacizumab. This period also marked the nascent exploration of cancer stem cells, which laid the groundwork for subsequent investigations. Research conducted during this era was predominantly foundational, aiming to establish a preliminary understanding of the ovarian cancer TME and to provide a direction and framework for future studies.

Between 2013 and 2020, the research focus progressively transitioned towards an in-depth examination of the interaction mechanisms among cells within the TME, the relationship between cancer stem cells and the TME, and the mechanisms underlying drug resistance. Concurrently, significant attention was directed towards immunotherapy and combination therapy. The application of immune checkpoint inhibitors in the treatment of ovarian cancer has become an important research direction. In recent years (2021-2024), immunotherapy has persisted as a prominent area of scholarly interest. Novel therapeutic approaches, such as photodynamic therapy and CAR-T cell therapy, have also begun to emerge within this domain. As research into the TME has advanced, the tumor immune microenvironment (TIME) has increasingly become a focal point of study. Additionally, the recent emergence of keywords such as lipid metabolism, machine learning, scRNA-Seq, and mechanotransduction may signify the forefront of research in this field.

To explore emerging research topics in the field, this study conducted a sudden citation analysis and obtained the top 50 references with the strongest citation bursts, of which 17 were in the state of citation sudden (**Figure 8**). We analyzed representative studies that may focus on the following areas. The first field is the single-cell level research on ovarian cancer. Two studies (27, 29) used scRNA-Seq technology. Hornburg et al. ‘s research (29) focuses on the single-cell analysis of the TME. Through single-cell sequencing, the cellular components and interaction mechanisms of ovarian cancer immunophenotype formation were deeply analyzed, including tumor cell characteristics, immune cell composition, and intercellular communication mechanisms under different immune infiltration modes. The research of Izar et al. (27) comprehensively described the single-cell landscape of the high-grade serous ovarian cancer ascites ecosystem, including cell composition, functional program differences, and anti-tumor effects of related pathway inhibition. This study deeply understands the biological characteristics of ovarian cancer from the perspective of single cells. The second domain involves studying the mechanisms and effectiveness of ICIs. Matulonis et al. (26) evaluated the efficacy and safety of Pembrolizumab (a PD-1 inhibitor) in patients with advanced recurrent ovarian cancer, and found that the expression level of PD-L1 was related to the efficacy. Mariathasan et al. (30) discussed the role of TGFβ signal in the TME, especially how it can weaken the response to PD-L1 blockade by limiting T cell infiltration. Jiang et al. (95) predicted the response of immune checkpoint blocking (ICB) treatment by developing the calculation method of tumor immune dysfunction and exclusion (TIDE), and emphasized the influence of T cell dysfunction and exclusion on the response of cancer immunotherapy. The third area involves using PARP inhibitors and other medications to treat ovarian cancer. González-Martín et al. (34) and Ray-Coquard et al. (31) respectively evaluated the efficacy of Niraparib and Olaparib in the treatment of advanced ovarian cancer, and further explored the effects of these drugs on the progression-free survival of patients under different conditions (such as whether there is a homologous recombination defect, BRCA mutation state, etc.), which provided important data support for the optimization of clinical treatment plans for ovarian cancer.

Generally speaking, the research topics of these studies mainly focus on the single cell analysis of the ovarian cancer TME, the efficacy and mechanism of ICIs, the study of new therapeutic drugs and their combined therapeutic effects, the mechanism of drug resistance, and the related research of TAMs. Interestingly, we found that these emerging research topics are almost consistent with the research hotspots in this research field. This phenomenon may need to be interpreted from many aspects and angles. First of all, the study of the TME in ovarian cancer remains in its nascent stages. During this phase, several pivotal research directions have been identified, each possessing significant scholarly value. Consequently, research hotspots and emerging research topics may focus on these key directions. For instance, immunotherapy in ovarian cancer represents a prominent area of interest, and the exploration of new immunotherapy targets or combined treatment schemes has become an emerging topic. Both of them are essentially around the general direction of immunotherapy. This continuous focus shows that the field is still digging deep into the details of these key themes, and there has not been a large-scale direction shift. Second, the application of new technology in this field is an important factor leading to this phenomenon. For instance, the advent of single-cell sequencing technology offers an unprecedented high-resolution perspective for examining the TME of ovarian cancer (74). This advancement has catalyzed a surge in research at the single-cell level. The research continuity promoted by this technology makes emerging topics and research hotspots closely linked. New technology is like a powerful engine, which drives a series of related research, from the preliminary exploration of hot issues to the deep excavation of emerging topics, all of which run on this technology-driven track. Third, ovarian cancer poses a significant threat to women’s health, necessitating an urgent demand for effective clinical treatments. Currently, enhancing the prognosis of ovarian cancer patients remains a critical objective in research. Consequently, research areas directly associated with clinical interventions, such as identifying more effective therapeutic targets and assessing the efficacy and safety of novel pharmaceuticals, have emerged as prominent and burgeoning topics within the field. Furthermore, the research on ICIs in the treatment of ovarian cancer is a hot field, and further exploring how to overcome the drug resistance of immunotherapy may become a new topic, which is to meet the actual needs of clinical treatment of ovarian cancer.

### 4.4 Limitations

Our analysis relies exclusively on the WoSCC. While the WoSCC is recognized as an authoritative and extensively utilized resource for bibliometric research, renowned for its rigorous journal selection and citation indexing, it does not encompass the entirety of scientific literature. Consequently, our study may have overlooked pertinent articles indexed in other prominent databases, such as Scopus or PubMed, particularly those originating from regional journals or specific publishers not included in the WoSCC. This limitation may introduce a bias in our findings, favoring journals predominantly indexed in the WoSCC with high impact factors, potentially underrepresenting contributions from certain regions or disciplines. Nevertheless, the WoSCC remains a respected standard for large-scale bibliometric analysis due to its extensive coverage of high-impact journals, detailed citation data, and user-friendly data structure, which facilitates the use of tools like CiteSpace and VOSviewer. Consequently, the research trends and focal areas identified in this study are deemed reliable, as the data sourced from the WoSCC adequately represents a substantial portion of the relevant information (96).

Certain forms of grey literature, including unpublished research findings, can constitute valuable contributions to this field. Nevertheless, the quality, accessibility, and data integrity of grey literature vary significantly, posing challenges for bibliometric analysis and potentially undermining its reliability in identifying stable research frontiers and key contributors. Furthermore, since these documents are not typically subject to citation or co-citation, they lack the structured data necessary for recognition by bibliometric software. The primary objective of bibliometric analysis is to construct a published and citable body of peer-reviewed knowledge. Consequently, by focusing on peer-reviewed publications, we ensure greater reliability and reproducibility of the core dataset.

Moreover, bibliometric analysis is inherently dependent on data from published literature, which can introduce a time lag effect. This effect arises because recently published works may not yet have accrued sufficient citations, thereby impeding an accurate assessment of their influence and significance. To solve this issue, we employ an additional method less susceptible to time lag: the citation burst analysis. This approach involves identifying and capturing emerging topics by detecting documents that exhibit a rapid increase in citations over a short period.

## 5 Conclusion

To further highlight the practical application value of this study, we especially emphasize its guiding significance for research funding and policy formulation. Firstly, this study reveals the key research directions and emerging topics within the TME of ovarian cancer, such as the optimization of immunotherapy, the application of single-cell technology, and the in-depth analysis of TME heterogeneity. This information provides clear guidelines for funding agencies to allocate funds, so that they can support projects with potential and influence more accurately and promote scientific breakthroughs and clinical transformation. The findings of this study can assist funding bodies in prioritizing projects that possess a robust scientific foundation, research value, and potential. This includes supporting comprehensive research on the factors influencing the efficacy of ICIs and exploring initiatives aimed at modifying the TME to enhance the effectiveness of immunotherapy. Through co-citation and citation burst analysis, this study has identified the research direction with significant clinical transformation potential in this field. These directions include the optimization of the therapeutic efficacy of ICIs in combination therapies and the targeted intervention of the TME immunosuppression mechanisms. Simultaneously, the application of single-cell sequencing technology provides a tool to deeply understand the cell interaction and heterogeneity in TME, and is helpful to find new immunotherapy targets. Furthermore, the study elucidates the distribution of research capabilities and collaborative networks within this domain, providing funding bodies and policymakers with insights to optimize resource allocation, enhance international cooperation, prevent redundant research efforts, and ultimately improve research efficiency and innovation capacity.

Ovarian cancer is characterized as a highly heterogeneous malignant neoplasm, with its TME playing a crucial role in the disease’s onset, progression, and therapeutic response. This study employs bibliometric analysis and visualization techniques to conduct a comprehensive examination of research trends and focal points within the context of the ovarian cancer TME. The study elucidates significant research directions in this domain and identifies the leading countries, institutions, and researchers, as well as those researchers and journals with high citation frequencies. Moreover, several prominent research hotspots have been identified, including ICIs, scRNA-Seq technology, the TME heterogeneity, the TGFβ signaling pathway, the impact of PARP inhibitors on the TME, mechanisms of drug resistance, and related studies on TAMs. These research hotspots not only reflect the focus of current academic circles but also provide a new direction for future research. With the application of new technology and the innovation of treatment methods, we are expected to make a breakthrough in the treatment of ovarian cancer and improve the prognosis of patients with ovarian cancer. Future research should continue to focus on these areas of research to advance the development and advancement of ovarian cancer treatment.

## Data Availability

The original contributions presented in the study are included in the article/supplementary material, further inquiries can be directed to the corresponding author/s.
