# Supplementary figures and images for: Research trends and hotspots in the tumor microenvironment of ovarian cancer: a bibliometrics and visualization study from 2005 to 2024
Source: Front Immunol. 2025 Aug 28;16:1605695. doi: 10.3389/fimmu.2025.1605695 (PMC12426531; doi:10.3389/fimmu.2025.1605695)

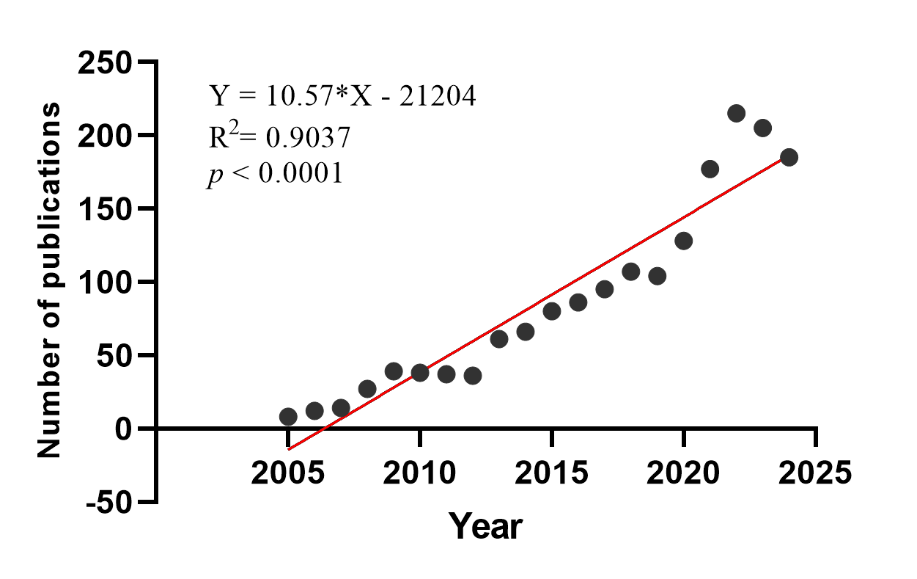


**Figure S1.** Fitting equation of annual publication volume.

Supplement: Supplementary file 2 [file DataSheet2.docx]
